# How political partisanship can shape memories and perceptions of identical protest events

**DOI:** 10.1371/journal.pone.0259416

**Published:** 2021-11-22

**Authors:** Eden Hennessey, Matthew Feinberg, Anne E. Wilson

**Affiliations:** 1 Department of Psychology, Wilfrid Laurier University, Waterloo, Ontario, Canada; 2 Rotman School of Management, University of Toronto, Toronto, Ontario, Canada; Sogang University (South Korea), REPUBLIC OF KOREA

## Abstract

It is well-recognized that increasingly polarized American partisans subscribe to sharply diverging worldviews. Can partisanship influence Americans to view the world around them differently from one another? In the current research, we explored partisans’ recollections of objective events that occurred during identical footage of a real protest. All participants viewed the same 87-second compilation of footage from a Women’s March protest. Trump supporters (vs. others) recalled seeing a greater number of negative protest tactics and events (e.g., breaking windows, burning things), even though many of these events did not occur. False perceptions among Trump supporters, in turn, predicted beliefs that the protesters’ tactics were extreme, ultimately accounting for greater opposition to the movement and its cause. Our findings point to the possibility of a feedback loop wherein partisanship underlies different perceptions of the exact same politically relevant event, which in turn may allow observers to cling more tightly to their original partisan stance.

## Introduction

Research shows that Americans are polarized in terms of their political views [1, 2], morality [3], and even the popular culture they consume. It is clear the two sides of the aisle have different worldviews, but can partisanship even lead people to view the facts of the world around them differently? In the present research we examine whether partisanship plays a role in shaping even visual perceptions of objective, politically relevant events, influencing individuals from opposing parties to observe the same set of events yet come away with different memories of what occurred, predicting diverging impressions of the event. Specifically, we explore how opposing partisans perceive the same footage of the Women’s March–and consider how these perceptual differences predict activist movement support in ways that could sustain or even exacerbate polarization and enflame the culture wars.

According to previous research, perceptions are motivated [4], and contribute to inaccurate memory recall and stereotype maintenance [5–8], which is not surprising given people are motivated to interpret information directionally, that is, if it confirms existing beliefs, they will incorporate the information, whereas if it contradicts existing beliefs, interpretation will be guided by existing beliefs. In a political context, partisans are apt to engage in directionally motivated reasoning [9, 10], leading them to seek out information that reinforces preferences (i.e., confirmation bias), and to counter-argue information that contradicts preferences (i.e., disconfirmation bias; [11]).

Perceptions of physical states and features of the environment can be motivated as well [12–14]–a perceptual bias that can occur as a result of political partisanship [15–19]. Along these lines, Kahan [20] proposed a politically motivated reasoning paradigm, which highlights how one’s political affiliation can lead people to perceive, interpret, and come to very different conclusions about the same event. One recent study strikingly demonstrated that committed Trump supporters, when shown Obama and Trump’s inauguration crowd, judged Trump’s obviously smaller inauguration crowd as the larger of the two [21]. The current study extends past research by assessing partisan perceptions of an identical protest event and examining how event memories that diverge from what objectively occurred can inform observers’ assessments of the extremity of the protest, which in turn predicts support for or rejection of the movement.

### The present research

In the present research we examined how partisanship affected perceptions of the Women’s March. The Women’s March was a worldwide protest event prompted largely by Donald Trump’s presidential inauguration. Notably, it was both one of the largest–and most peaceful–protests in recent history [22]. This is relevant in light of evidence that extreme protest behavior, although it garners attention, also risks decreasing support for a social movement and the movement’s cause [23]. Might the Women’s March, with its peaceful and positive atmosphere, be immune then from this risk? We suspected that good behavior might still be no match for partisan perception: partisan opponents might still perceive the protest in ways that confirm their disapproval, even in the objective absence of antisocial events. We presented all participants with a video clip compiled from footage of the protest event (which was peaceful and non-violent) and then assessed participants’ perceptions about what did and did not happen in the video.

Overall, we hypothesized that Trump supporters would falsely perceive that the protesters engaged in negative and extreme behaviors (e.g., fights, destroying property). Just as actual extreme behavior decreases public support for activists [23], we expected that *imagined* (i.e., misremembered) bad behavior would do the same even in the absence of any evidence. If partisan opponents can construe even a peaceful protest as extreme, then these misperceptions can justify rejection of the movement and its goals. The specific process that we test in the current research could reflect a broader phenomenon in which partisan polarization shapes people’s perceptions of the facts on the ground, which in turn could sustain, justify, or even intensify partisan animosity.

## Materials and methods

### Participants

This research was approved by the University of Toronto Research Ethics Board (University of Toronto Research Ethics Board #31102). Online consent was provided by clicking the appropriate option. We had no past research on which to estimate effect size, so aimed for a sample size that would allow for the detection of a relatively small effect (.2) across two partisan groups. G*Power 3.1 [24], indicated an adequate sample size of 314 with power set at .95 and alpha = .05 (two-tailed). Recognizing the possibility of exclusions and possibly unequal partisan groups, we recruited a sample larger than this recommended minimum. Participants (*N* = 420) were recruited from Mechanical Turk for a study on personality and attitudes. As previous research has shown, Mechanical Turk respondents tend to lean liberal [25]. However, comparisons between Mechanical Turk and national samples are largely comparable [26], suggesting that Mechanical Turk is a valid recruitment tool for research on political ideology. Those who either did not view the video (*n* = 28, as indicated by the timer) or did not answer questions about their recall of the video were removed from analyses (in all cases, most or all of the key dependent variables following the video were missing), resulting in a final sample of *n* = 351.

Recognizing that attrition rates could have varied by voter status (e.g., perhaps Trump voters would skip the video or discontinue the survey more often), we examined this possibility. Those who did versus did not watch the video did not significantly differ by voter status (*χ*^2^ (1, *N* = 420) = .007, *p* = .93). Those who completed the questions about the video and those who did not did not significantly differ by voter status (*χ*^2^ (1, *N* = 420) = 1.56, *p* = .21). On average, participants spent 108 seconds (ranging from 73.52 seconds to 710.63 seconds) on the survey page containing the 87-second video. Although we cannot guarantee that they watched the video closely, the time spent on the video page suggests that they viewed all or most of the video. Viewing time did not significantly differ by voting status (*p* = .058), with Trump viewers watching marginally longer.

Self-reported ethnicities were 78% White, 8.3% Black, 6.6% Hispanic, 6% Asian, and 1.1% Other. Self-reported genders were 52.1% male and 47.9% female, and the average age was *M*_*age*_ = 37.05, *SD* = 12.33. Participants were compensated with $1 USD.

### Procedure

Participants accessed the online survey and were first asked to provide demographic information such as age, gender, ethnicity, political ideology, and who they voted for (or would have voted for) in the 2016 Presidential election. Participants then watched a short video and answered questions about the video content and perceptions of the movement and its members.

### Protest event video clip

Participants watched a video montage from the Women’s March on Washington that occurred on January 21, 2017. The video clip was sourced from an online group and edited to remove corporate affiliations. The footage was 87 seconds long and included multiple scenes of different crowds chanting, holding signs, and marching (see Supporting information for link to video). Before the video, participants read these instructions:

In the next part of the study, you will be randomly assigned to watch one of several different videos of protest events. As you watch the video, pay very close attention to what you see and hear, as we will ask you detailed questions about the clip afterwards.

When the video finished, participants were asked various questions about the video’s content. Two independent raters coded the video clip to establish the number of actual events that occurred within the footage. There was 100% agreement on objective events that did not occur. Agreement was very high for events that did happen, with only minimal differences for a few frequent categories (for instance, estimated number of pussy hats were both over 100 but varied by a few hats). These responses were averaged across the two raters.

### Measures

#### Predictors

*Political ideology*. Participants indicated their political ideology on a scale from (1) *Extremely Liberal* to (7) *Extremely Conservative*.

*Voter status*. Participants indicated who they voted for (those who did not vote but identified who they would have voted for were also included) in the American November 2016 Presidential election. Response options included Donald Trump, Hillary Clinton, Gary Johnson, Jill Stein, or other. Voting percentages were as follows: 46.4% Hillary Clinton, 31.1% Donald Trump, 9.1% Unspecified, 5.4% Gary Johnson, 3.1% Bernie Sanders, 2.8% Jill Stein, 2.1% other. Responses were dummy-coded such that those who voted for Donald Trump were coded “1”, and those with other responses were coded “0”. Results were consistent if only Trump and Clinton voters were compared and if those with unspecified voting intentions were included. We focus on Trump-supporters versus others instead of political ideology because Trump supporters, especially right after the 2016 election, did not always fit into the traditional liberal–conservative divide. Even so, the results of political ideology closely mirror the results reported here regarding Trump supporters (vs. others). See (S1 Table in S2 File) for details.

#### Outcomes

After watching the video clip, participants were asked to report perceptions of protesters and to indicate the number of times they saw various events in the video.

#### Count variables

We created a set of items and actions that could objectively be identified as visibly present at the protest, as well as items and actions that objectively were not visibly present. We included objectively absent items that would reflect negatively on protesters, as well as some objectively absent items that were relatively neutral.

*Negatively-valenced false events*. Participants were asked to indicate how many times they saw nine items that were not actually in the footage and were negatively-valenced across a range of more or less offensive events: people wearing masks, burning things, smoking marijuana, breaking windows, in fights or brawls, with exposed breasts, holding signs or chanting “burn it down!,” Mexican flags, and signs with misspelled words. Items were tested as composites and individual outcomes. We grouped Mexican flags into negatively-valenced items because media indicates that this flag is politically charged, though we acknowledge its valence would vary depending on partisanship [27, 28].

*Neutral false events*. Participants were asked to indicate how many times they saw two items that were not actually in the footage, but were neutrally-valenced: animals or pets, and signs or posters with cartoons.

*Actual events*. Participants were asked to indicate how many times they saw four items that appeared in the video footage in varying quantities: people wearing pink ‘pussy’ hats, American flags, signs or posters with references to Donald Trump, and signs or chanting with curse words.

#### Continuous variables

*Protest tactics*. Participants rated the extent to which they perceived the protesters as employing various positive and negative protest tactics. Positive tactics were measured by creating a composite score out of four items: “To what extent were the protesters expressing positive emotion (e.g., happiness, pride, love)?”, “To what extent were the protesters chanting or holding posters that used humor?”, “To what extent were the protesters chanting or holding posters that emphasized kindness and goodwill?”, “To what extent were the protesters chanting or holding posters that used creativity (e.g., rhymes, puns)?” Items were rated on a scale from (1) *Not at All* to (5) *A Great Deal*. The measure showed acceptable reliability (α = .617).

Negative tactics were measured by creating a composite score out of five items: “To what extent were the protesters expressing negative emotion (e.g., anger, frustration, outrage)?”, “To what extent were the protesters saying mean things about Donald Trump as a person?”, “To what extent were the protesters making fun of Donald Trump as a person?”, “To what extent were the protesters chanting or holding posters that emphasized antisocial behavior?” and, “To what extent were the protesters chanting or holding posters that used rude or offensive language?” Items were rated on a scale from (1) *Not at All* to (5) *A Great Deal*. The measured showed good reliability (α = .833).

*Support for the movement*. Support for the movement was measured with a composite of five items rated on a scale from (1) *Not at All* to (5) *Very Much*: “Overall, how much do you support members of this movement?”, “Overall, how much do you support this movement’s cause?”, “How willing or unwilling would you be to join these activists at a protest event?”, “How much do you feel the members of this movement are similar to you?**”** and, “How much do you identify with the members of this movement?” The measure showed excellent reliability (α = .951).

*Perceived extremity*. Participants rated the single item: “To what extent would you say the protesters’ behavior was extreme?” on a scale from (1) *Not at All* to (5) *Very Much*.

## Results

Descriptive statistics and intercorrelations for continuous variables appear in Table 1.

**Table 1:** Descriptive statistics and intercorrelations for continuous variables.

Continuous Outcome Variables	1	2	3	4	Means (*SD*)
**1. Positive protest tactics**	---	-.137*	-.197**	.525**	2.77 *(*.*776)*
**2. Negative protest tactics**	---	---	.598**	-.196**	2.10 *(*.*852)*
**3. Perceived extremity**	---	---	---	-.309**	1.81 *(1*.*08)*
**4. Support for the movement**	---	---	---	---	2.92 *(1*.*28)*

*Notes*.

*p < .05

** *p* < .001.

Participants’ self-reported political ideology was just below the scale midpoint (*M* = 3.55, *SD* = 1.79) indicating a mild liberal lean. Most participants (68.9%) indicated support for other candidates in the 2016 Presidential election, whereas 31.1% indicated support for Donald Trump. The dummy-coded variable for voters (Trump = 1, others = 0) was used as the partisanship independent variable. First, we examine differences between Trump supporters and other supporters on continuous variables using t-tests. Then, we present comparisons of the counts of events and non-events (i.e., frequencies), using chi-square, zero-inflated count, and Tobit regression analyses, across Trump supporters versus other supporters. Finally, we present a mediation model in which Trump supporters versus other supporters perceived more negative false events, that predicted greater perceptions of extremity and lowered support for the movement.

### Comparing Trump supporters vs. other supporters

Descriptive statistics and t-tests for all comparisons of variable means appear in Table 2.

**Table 2:** T-tests between Trump supporters and other supporters on key outcome measures.

	Voter Group					
	Trump		Other		Independent Samples	Effect size (Hedge’s *g)*
	(*n* = 109)		(*n* = 242)		T-tests; 95% CI	
**Continuous Outcome Variables**	Mean	*SD*	Mean	*SD*		
**1. Extremity** *	2.25	1.23	1.61	.95	*t* (168.29) = 4.84,	.61
					*p* < .001,	
					95% CI [.38, .90]	
**2. Negative tactics** *	2.36	.97	1.98	.77	*t* (171.14) = 3.62,	.45
					*p* < .001,	
					95% CI [.17, .59]	
**3. Positive tactics**	2.54	.84	2.87	.73	*t* (349) = -3.82,	.43
					*p* < .001,	
					95% CI [-.51, -.16]	
**4. Support for the movement** *	2.03	.91	3.32	1.21	*t* (270.93) = -11.03,	1.15
					*p* < .001,	
					95% CI [-1.52, -1.06]	
**Count Outcome Variables**						
	Mean	*SD*	Mean	*SD*		
**Sum false recall negative events** *	1.61	2.72	.897	1.72	*t* (148.28) = 2.50,	.34
					*p* = .013,	
					95% CI [.15, 1.27]	
**Sum false recall neutral events**	.697	.70	.703	.68	*t* (348) = -.066,	.009
					*p* = .947,	
					95% CI [-.16, .15]	
**Sum recall true events**	1.98	1.37	2.16	1.26	*t* (348) = -1.17,	.14
					*p* = .24,	
					95% CI [-.47, .12]	

*Notes*.

*denotes a significant Levene’s Test for Equality of Variances. When Levene’s test was significant, the (reduced) degrees of freedom and statistics are reported for *equal variances not assumed*Composites of remembered events are calculated as a sum of all recalled yes/no occurrences (coded as 0 = no occurrence, 1 = at least 1 recalled occurrence).

Sum false recall negative events (9 total): masks, burning things, breaking windows, smoking marijuana, exposed breasts, fights/brawls, “burn it down” signs, Mexican flags, misspelled signs

Sum false recall neutral events (2 total): pets/animals, signs with cartoons

Sum recall true events (4 total): pink “pussy” hats, Trump references, signs with curse words, American flags

Because sample size differs by voter group, Hedge’s *g* is reported for effect size. Effect size for Hedge’s g interpreted similar to Cohen’s d; small .2, medium .5, large .8

### Continuous variables

Independent samples comparisons of variable means showed significant differences between voter groups on all continuous self-report measures (see Table 2). Specifically, Trump supporters perceived the behavior in the video clip as more extreme than other supporters. Similarly, Trump supporters perceived significantly greater use of negative tactics and less use of positive tactics compared to other supporters. Finally, Trump supporters reported significantly lower support for the movement than other supporters.

### Count variables

Table 2 also reports aggregated occurrence scores for specific event types (negative false events, neutral false events, neutral real events). Because false event memories contained a high proportion of zeros and a positive skew (where most people who reported observing the non-existent false events reported a small number, but a few reported a large number of such events), we took a few analytic approaches to address the non-normal distribution (Tables 2 and 3). First, we summed an “occurrence count” of each event type where people who reported seeing at least one such event were coded as 1 and those who did not report seeing the event were coded as zero. These aggregates are reported in Table 2. T-tests showed that Trump supporters (vs. other supporters) perceived a significantly greater number of instances of negative false events (events reported to have occurred that objectively did not happen), including more people burning things, breaking windows, exposing their breasts, and holding signs with spelling errors (Table 2). In contrast, Trump supporters and other supporters perceived no significant differences in the perceived occurrence of neutral false events in the video clip (i.e., pets or animals, signs with cartoons) nor in the overall occurrence of actual events (events that objectively did happen), including people wearing pink “pussy” hats, making Trump references, signs with curse words, and American flags.

**Table 3:** Chi-square and regression analyses for participants’ estimated frequencies of false events in the video clip.

Estimated Frequencies on Count Variables (False Events)	None		= />1		Analysis		
Supporters	Trump	Other	Trump	Other	Chi-Square	Zero Inflated Count	Tobit Regression
	%	%	%	%			
***Negatively-valenced false events***							
**1. Wearing masks**	72.5	78.5	27.5	21.5	*X*^*2*^ (1) = 1.53, ϕ = .07, *p* = .216	*b* = -.33, *SE* = .27, *p* = .218	*b* = 7.33, *SE* = 4.82, *p* = .128
**2. Burning things**	86.2	94.2	13.8	5.8	*X*^*2*^ (1) = 6.31, ϕ = .13, *p* = .012	*b =* -.95, *SE* = .39, *p* = .015	*b =* 4.39, *SE* = 1.93, *p* = .023
**3. Breaking windows**	86.2	94.6	13.8	5.4	*X*^*2*^ (1) = 7.14, ϕ = .14, *p* = .008	*b =* -1.01, *SE* = .40, *p* = .011	*b =* 5.61, *SE =* 2.31, *p =* .015
**4. Smoking marijuana**	90.8	95	9.2	5	*X*^*2*^ (1) = 2.27, ϕ = .08, *p* = .132	*b* = -.66, *SE* = .45, *p* = .137	*b =* 6.17, *SE* = 4.56, *p =* .176
**5. Exposed breasts**	90.7	97.5	9.3	2.5	*X*^*2*^ (1) = 7.87, ϕ = .15, *p* = .005	*b* = -1.40, *SE* = .54, *p* = .009	*b* = 3.76, *SE* = 1.63, *p* = .021
**6. Fights or brawls**	88.1	95	11.9	5	*X*^*2*^ (1) = 5.52, ϕ = .13, *p* = .019	*b* = -1.05, *SE* = .43, *p* = .014	*b =* 3.03, *SE* = 1.58, *p* = .055
**7. Signs “burn it down!”**	80.7	87.2	19.3	12.8	*X*^*2*^ (1) = 2.48, ϕ = .08, *p* = .115	*b* = -.52, *SE* = .32, *p* = .107	*b =* 1.30, *SE* = .92, *p =* .161
**8. Mexican flags**	80.7	88.4	19.3	11.6	*X*^*2*^ (1) = 3.71, ϕ = .10, *p* = .054	*b* = -.79, *SE* = .33, *p* = .017	*b* = 2.19, *SE* = 1.78, *p* = .219
**9. Signs misspelled**	63.3	79.7	36.7	20.3	*X*^*2*^ (1) = 10.60, ϕ = .17, *p* = .001	*b* = -1.00, *SE* = .28, *p <* .001	*b* = 1.94, *SE* = .82, *p* = .019
***Neutral false events***							
**1. Pets or animals**	86.2	86.8	13.8	13.2	*X*^*2*^ (1) = .019, ϕ = .01, *p* = .891	*b* = -.01, *SE* = .34, *p* = .970	*b =* .42, *SE =* 1.04, *p* = .688
**2. Signs w/ cartoons**	43.5	43	56.5	57	*X*^*2*^ (1) = .009, ϕ = -.01, *p* = .924	*b* = .04, *SE* = .24, *p* = .857	*b* = 1.98, *SE* = 1.62, *p* = .222

*Notes*. Regression analyses were conducted using Winsorized data to account for outliers.

### Additional analyses for negative and neutral false events

Chi-square and regression analyses appear in Table 3. Prior to conducting analyses, numeric responses for negative false events and neutral false events were recoded into dichotomous outcomes, such that values of zero remained zero, and responses from 1 through to the highest value were recoded as 1 (Table 3). We also coded numeric responses for negative and neutrally-valenced false events into three categories, such that responses of zero remained zero, responses 1–10 were recoded as 1, and responses 11 to the highest value were coded 2. Chi-square analyses testing supporter group differences using this trichotomous coding appear in (S2 Table in S2 File), indicating a similar pattern of results to the dichotomous analyses. Chi-square analyses tested the proportion of reported occurrence/non-occurrence for each item for Trump- and other-supporters.

Given that the false events did not occur in the video at all, most respondents reported zero for these events. As a result, we also conducted zero-inflated and Tobit regression analyses to account for zero-inflation and outliers (Table 3). We presented these additional analyses to give the reader clearer information about the robustness of effects using a variety of methods. Results are virtually identical across three analysis types with the exception of Mexican flags (which was significant in only one of three analyses).

Overall, Trump supporters perceived a greater number of negative false events in the video clip than other supporters for five of the eight negative events. Specifically, when asked how many times they had seen people in the clip burning things, breaking windows, exposing their breasts, misspelled signs, and fighting, Trump supporters were more likely than other supporters to report seeing at least one incident, although they did not occur in the clip even once. There were no significant differences between groups for three negative events: people holding signs saying, ‘burn it down!’, people wearing masks, or seeing people smoking marijuana. There were no significant differences between Trump supporters and other supporters on neutral false events (i.e., pets or animals, signs with cartoons).

### Count analyses for actual events

For false events, reported above, accuracy is determined my simply examining the number of reports of non-occurrence (the accurate response) versus occurrence (inaccurate). To test the accuracy of participants’ perceptions of actual events in the video clip, we can compare reported frequency of events with their actual observed frequency. We conducted one sample t-tests of actual event reported counts against the actual values (how many actual “pussy” hats, American flags, references to Trump, and curse words were observed in the video, averaged across two raters) within groups of Trump supporters and other supporters (Table 4).

**Table 4:** Chi-square and regression analyses for participants’ estimated frequencies of actual events in the video clip.

Estimated Frequencies on Count Variables	None		= />1					
Supporters	Trump %	Other %	Trump %	Other %	Chi-Square	Actual #	One Sample T-tests	
							Trump Supporters	Other Supporters
**1. Pink ‘pussy’ hats**	37.6	31.8	62.4	68.2	*X*^*2*^ (1) = 1.13, ϕ = -.06, *p* = .287	106.5	*M* = 17.20,	*M* = 21.58,
							*SD =* 35.89	*SD =* 41.14
							*t* (108) *=* - 25.97,	*t* (241) *= -*32.11,
							*p <* .001	*p <* .001
**2. Trump references**	52.3	40.1	47.7	59.9	*X*^*2*^ (1) = 4.55, ϕ = -.11, *p* = .033	3.5	*M* = 2.72,	*M* = 4.01,
							*SD =* 7.05	*SD =* 7.63
							*t* (108) *=* -1.16,	*t* (241) *=* 1.04,
							*p =* .248	*p =* .301
**3. Signs w/ curse words**	56.9	66.9	43.1	33.1	*X*^*2*^ (1) = 3.30, ϕ = .10, *p* = .070	4	*M* = 1.90,	*M* = 1.15,
							*SD =* 3.61	*SD =* 2.53
							*t* (108) *=* - 6.07,	*t* (241) *=* -17.54,
							*p <* .001	*p <* .001
**4. American flags**	55	45.5	45	54.5	*X*^*2*^ (1) = 2.77, ϕ = -.09	9	*M* = 1.24,	*M* = 2.05,
					*p* = .096		*SD =* 1.91	*SD =* 3.13
							*t* (108) *=* - 42.44,	*t* (241) *=* -34.54,
							*p <* .001	*p <* .001

*Notes*. Chi-square analyses indicate group differences between Trump vs. other supporters. One sample t-tests refer to the accuracy of perceptions within each groups of supporters compared to the actual number of events. Actual values reflect an average across two independent raters.

Analyses revealed that participants were somewhat accurate in estimating the number of actual references to Donald Trump (i.e., 3.5) in the video clip (other supporters: *M* = 4.01, *SD* = 7.63; Trump supporters: *M* = 2.72, *SD* = 7.05). However, participants were less accurate in estimating the number of times they saw signs with curse words; other supporters (*M* = 1.15, *SD* = 2.53) and Trump supporters (*M* = 1.90, *SD* = 3.61) perceived significantly fewer instances of signs with curse words than were in the video clip (i.e., 4). Similarly, other supporters (*M* = 2.05, *SD =* 3.13) and Trump supporters (*M* = 1.24, *SD =* 1.91) reported seeing significantly fewer American flags than were really in the video clip (i.e., 9). Finally, other supporters (*M* = 21.58, *SD =* 41.14) and Trump supporters (*M* = 17.20, *SD =* 35.89) alike reported seeing significantly fewer pink ‘pussy hats’ than were in the video clip (i.e., 106.5). In sum, both Trump supporters and other supporters were largely inaccurate in their perceptions of the number of actual events in the video clip. However, Trump supporters and other supporters did not differ from one another in their estimates of actual events with the exception of Trump references (which were recalled significantly more frequently by other supporters).

### Mediation analyses

#### Serial mediation model

**Fig 1:**
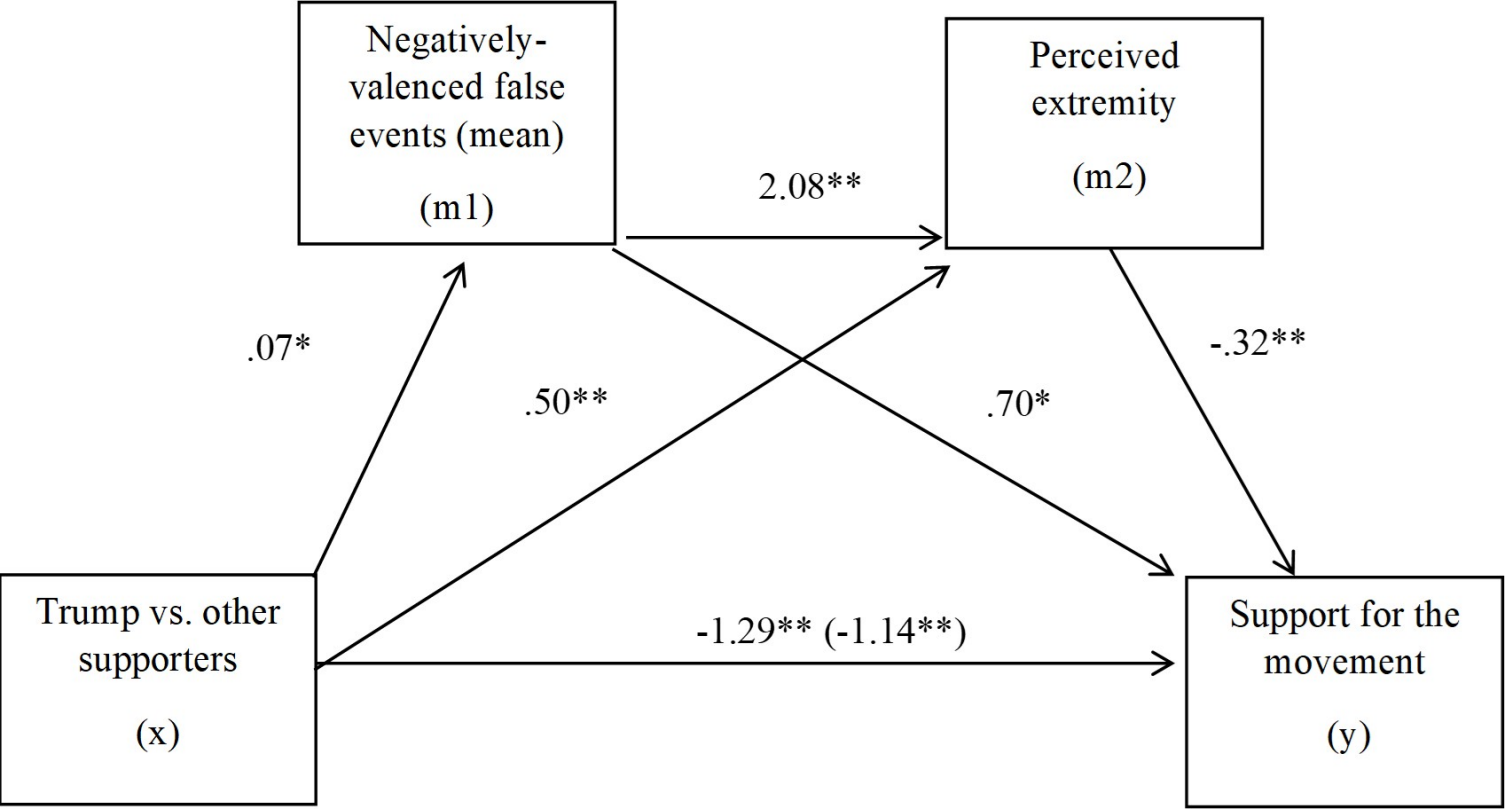
Serial mediation model. This figure shows a serial mediation model in which Trump supporters versus other supporters recalled more negatively-valenced false events in the video clip, which was associated with greater perceptions of extremity and lowered support for the movement. * *p* < .05, ** *p* < .001.

Recall that we expected Trump supporters (vs. other supporters) to perceive a greater total number of negatively-valenced false events (e.g., burning things, breaking windows), which would in turn predict increased perceptions of extremity and ultimately, lowered support for the movement. Hayes’ [29] PROCESS macro Model 6 was used to test the serial mediating effects of the mean of perceived negatively-valenced false events and perceived extremity in the relation between voter status and support for the cause. Indirect effects were tested using a bootstrap estimation approach with 5,000 samples. The indirect effect of voter status on support for the movement through perceiving negatively-valenced false events and extremity was significant, *b* = -.04, *SE* = .03, 95% CI [-.10, -.004], illustrating that for Trump supporters, support for the movement was partially mediated and reduced through perceiving a greater number of negatively-valenced false events and greater extremity of the protest event (Fig 1).

Notably, the link between negatively-valenced false events and perceived extremity was significant (*b* = 2.08, *p* < .001), suggesting that Trump supporters perceived the protest movement as extreme in part because they perceived negative events that did not occur. All indirect effects were significant. This model is also significant entering the mean of negative tactics as the first mediator instead of the mean of the negatively-valenced false events. Alternate mediation models appear in (S1 and S2 Figs).

## Discussion and conclusions

The present study assessed how partisanship motivated diverging (and often false) perceptions of a political protest event, and how these perceptual differences, in turn, predict support for or rejection of a political movement. As expected, perceptions of the event differed significantly according to partisanship, such that Trump supporters versus other supporters perceived more negative and fewer positive protest tactics, perceived the protest event as more extreme, and reported lower support for the movement. However, as with any motivated process, the strength of participants’ initial biases likely played a role in how strongly people engaged in motivated perception. For example, strength of initial political affiliation (e.g., support for Trump) could predict the degree to which participants misperceived events and came to more uncharitable conclusions about protesters. It is difficult to entirely disentangle causal processes, and because our mediation models are based on correlational data they must be interpreted cautiously with regard to causality. Indeed, some pathways in the mediation model were statistically weaker than others, perhaps because participants reported on negatively valenced false events in one path (i.e., from x to m1) versus a more ambiguous measure of perceived extremity (i.e., from x to m2).

We also found that perceptions of the numbers of events in the video clip differed according to voter status, such that Trump supporters (vs. others) reported seeing a greater number of negatively-valenced false events, such as people burning things or breaking windows. Notably, these negatively-valenced false events did not actually occur in the video clip, but Trump supporters (vs. others) were significantly more likely to report seeing instances of them occurring. Regarding people’s memory for actual events in the clip (i.e., events that really occurred), few differences emerged among voter groups, suggesting that participants were not motivated to see actual events differently, but only the (objectively non-existent) negatively-valenced events. Our findings therefore add to existing research demonstrating the powerful influence of partisan views on the ways in which information is processed and interpreted [15–20].

A major contribution of the present work is that we demonstrated how divergent perceptions of a protest, fuelled by partisanship, could contribute to sustaining or even intensifying polarized responses to that protest. Specifically, our findings showed that perceiving more negative tactics or negative events that did not occur, predicted perceptions of extremity, which in turn predicted lowered support for the movement. Thus, the current study moves beyond research demonstrating that partisanship fuels diverging perceptions of an actual event [21], to show how such perceptions predict seeing actions as more extreme, which ultimately predicted diminished support for the cause. The present findings therefore may point to a feedback loop in which partisanship predicts perceptions of political events that confirm the narrative that political opponents are bad or evil. The consequences of such differing perceptions of protest events can be severe. Instead of drumming-up support for the movement, if opponents are left with lasting memories of extremity and violence that never happened, even peaceful protests could be used by opponents to justify or bolster opposition to the movement.

The current findings also have fundamental implications for social movements. As in previous research describing the activist’s dilemma to use extreme tactics to gain attention but paradoxically turn off potential members by doing so [23], our results illustrate the potential perils of employing negative or extreme collective actions in response to perceived injustice. However, the current findings also suggest that regardless of the actual behaviors enacted by protesters, political partisanship will shape perceptions of protest events above and beyond reality. Thus, even peaceful protesters, seeking to present themselves as inclusive and non-threatening, might still be perceived as employing extreme protest tactics by a portion of observers.

One limitation of the present research is that we measured perceptions of a protest event that was liberal leaning. It would be useful for future research to replicate the current findings in the context of a conservative protest event, such as one related to firearm legislation or anti-abortion activism. In these cases, one could speculate that more liberal participants would falsely perceive more negative and fewer positive protest tactics, leading to increased perceptions of extremity and greater opposition for the movement and its cause, whereas the opposite pattern could be expected among more conservative participants. Absent evidence to the contrary, we would posit a symmetrical vulnerability to these perception processes; indeed, in a recent meta-analysis of studies on partisan bias, liberals (*r* = .235) and conservatives (*r* = .255) showed no significant difference in mean levels of bias across studies [30]. For example, we would expect Democrats to recall more egregious behaviour on the part of Republican anti-lockdown protesters or January 6, 2021 Capitol insurrectionists. An additional limitation of the current work is the extent to which we can conclude that false memories of misbehavior were genuinely recalled and not reported as a means of expressing political views [21]. Research has yet to compellingly disentangle the process of genuine motivated belief from the process of expressive belief, but arguably both can have similar implications for polarization.

In all, we suggest that the underlying worldview differences that divide individuals into partisan camps also impact how individuals literally view politically relevant events in the world around them. Even in the face of identical stimuli, people are apt to see what they want to, motivated by their own political narratives instead of factual accuracy. As a result, partisans perceive different realities which could in turn provide further fuel for the differences between the two sides.

## Supporting information

S1 Fig This parallel mediation model shows that those who voted for Trump (versus others) perceived more negative and fewer positive protest tactics, which in turn, predicted to seeing protest behaviors as more extreme. **p < .001, * *p* < .05.
(TIF)

S2 Fig This serial mediation model shows among Trump (versus others) supporters, perceiving more negative protest tactics in the video clip predicted greater perceptions of the protest as extreme, which predicted lowered support for the cause. **p < .001, *p < .05.
(TIF)

S1 File This document contains a link to online video montage, additional analyses, S1 and S2 Tables in S2 File, and a variable guide to facilitate working with the data and syntax files provided. (DOCX)

S2 File This SPSS data file contains variable needed to replicate analyses. (DOCX)

S1 Data This SPSS syntax file contains code for replicating analyses. (SAV)

S2 Data (SPS)
