# Supplementary figures and images for: How political partisanship can shape memories and perceptions of identical protest events
Source: PLoS One. 2021 Nov 22;16(11):e0259416. doi: 10.1371/journal.pone.0259416 (PMC8608305; doi:10.1371/journal.pone.0259416)

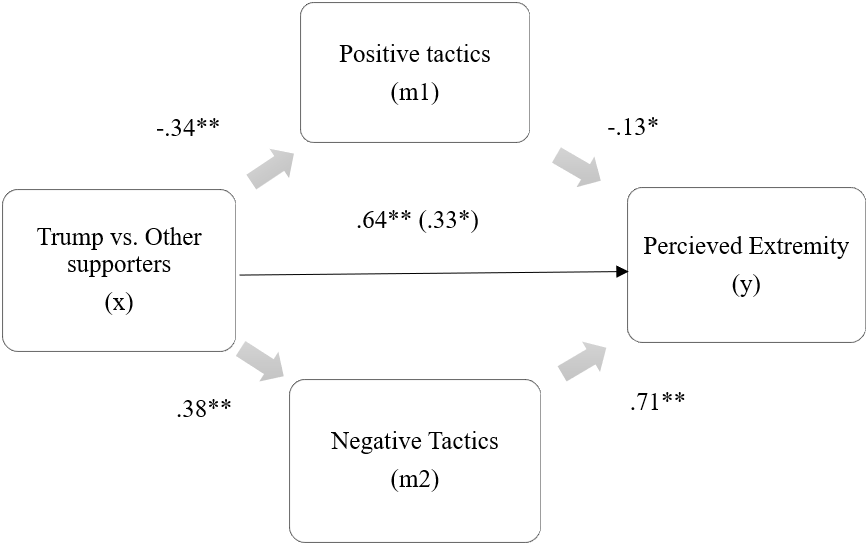

Supplement: S1 Fig — **p < .001, * p < .05. (TIF) [file pone.0259416.s001.tif]

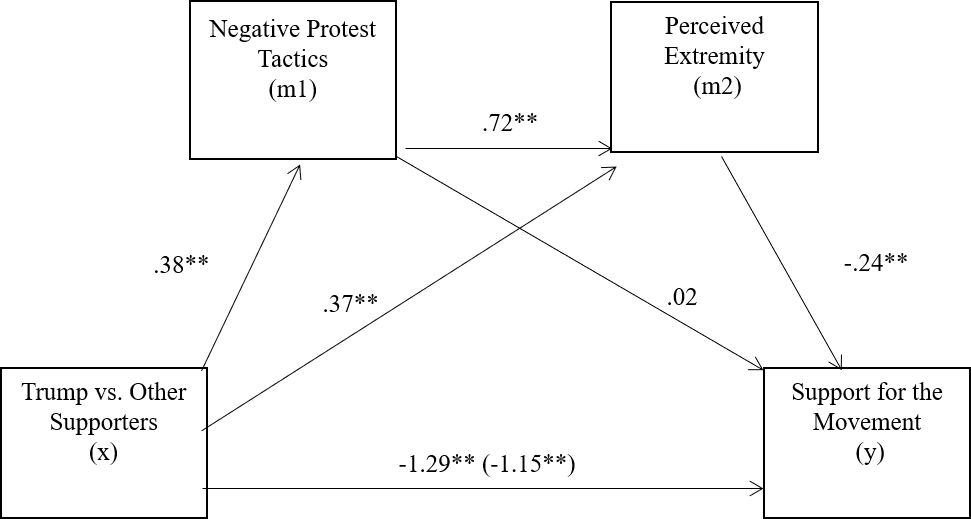

Supplement: S2 Fig — **p < .001, *p < .05. (TIF) [file pone.0259416.s002.tif]
