# Supplementary material for: How political partisanship can shape memories and perceptions of identical protest events
Source: PLoS One. 2021 Nov 22;16(11):e0259416. doi: 10.1371/journal.pone.0259416 (PMC8608305; doi:10.1371/journal.pone.0259416)
Supplement: S1 File — (DOCX) [file pone.0259416.s003.docx]

**PLOS One**

**Variable Guide**

**Hennessey, Feinberg, & Wilson (2021)**

*This document contains variables names and descriptions for all analyses.*

1. polID = continuous measure of political ideology

2. PositiveTactics_mean = mean of 4 items assessing the extent to which protesters used positive protest tactics like expressing positive emotion

3. NegativeTactics_mean = mean of 5 items assessing the extent to which protesters used positive protest tactics like expressing negative emotion

4. BehExtreme = single item assessing perceptions of extremity of the protesters’ behaviour

5. SupportID_mean = mean of 5 items assessing the extent to which participants support the protesters and the movement

6. NegFalse_SUM = sum of 9 items assessing number of objectively false negative events such as wearing masks and burning things

7. NegNeutral_SUM = sum of 2 items assessing number of objectively false neutral events such as pets

8. TrueEvents_SUM = sum of 4 items assessing number of objectively true events such as pink ‘pussy’ hats

9. MEAN.mismemory.R = objectively false negative events such as wearing masks and burning things

10. Count variables recoded into binary outcomes: 1 = incorrect, 0 = correct

Recall_items_masks.Rcorrect

Recall_items_burning.Rcorrect

Recall_items_breakwindows.Rcorrect

Recall_items_marji.Rcorrect

Recall_items_breasts.Rcorrect

Recall_items_fights.Rcorrect

Recall_items_signsburnitdown.Rcorrect

Recall_items_Mxflags.Rcorrect

Recall_items_signsepelling.Rcorrect

Recall_items_pets.Rcorrect

Recall_items_signcartoons.Rcorrect

Recall_items_pussyhats.Rcorrect

Recall_items_signsrefDT.Rcorrect

Recall_items_signcurses.Rcorrect

Recall_items_USflags.Rcorrect

11. Count variables Winsorized:

Recall_items_masksw

Recall_items_burningw

Recall_items_breakwindowsw

Recall_items_mariw

Recall_items_breastsw

Recall_items_fightsw

Recall_items_signsburnitdownw

Recall_items_Mxflagsw

Recall_items_signsspellingw

Recall_items_petsw

Recall_items_signscartoonsw

Recall_items_pussyhatsw

Recall_items_signsrefDTw

Recall_items_signscursew

Recall_items_USflagsw

12. Count variables recoded into trichotomous outcomes (0 = 0, 1-10 = 1, 11 to the highest value= 2)

Recall_items_masks.R

Recall_items_burning.R

Recall_items_breakwindows.R

Recall_items_marji.R

Recall_items_pussyhats.R

Recall_items_breasts.R

Recall_items_USflags.R

Recall_items_Mxflags.R

Recall_items_pets.R

Recall_items_fights.R

Recall_items_signcurses.R

Recall_items_signcartoons.R

Recall_items_signsrefDT.R

Recall_items_signsepelling.R

Recall_items_signsburnitdown.R
