# Supplementary material for: How political partisanship can shape memories and perceptions of identical protest events
Source: PLoS One. 2021 Nov 22;16(11):e0259416. doi: 10.1371/journal.pone.0259416 (PMC8608305; doi:10.1371/journal.pone.0259416)
Supplement: S2 File — (DOCX) [file pone.0259416.s004.docx]

**Supporting Information**

**Link to video montage:** <https://www.youtube.com/watch?v=RMii7j1alFU&list=PLf4t8S1QEJNwd2rlrsHU-Sw03JdJMBxqx>_++=_

Note that corporate affiliation was removed prior to use in the study.

We gratefully acknowledge Brookline Interactive Group for providing the video clip on YouTube.

**Additional Analyses**

**Linear Regression Analyses**

Political ideology was a significant predictor of perceptions of the protest event (See Table S1). Specifically, analyses revealed that greater conservatism was associated with perceiving more negative protest tactics and fewer positive protest tactics. Analyses also showed that greater conservatism was associated with perceiving the protest event as more extreme. Finally, greater conservatism was associated with decreased identification with the protesters and lowered support for the movement.

| **Table S1. Unstandardized Regression Coefficients for Political Ideology Predicting Outcomes** | | | | |
| --- | --- | --- | --- | --- |
| Outcome Measures | *B* | *SE* | *t* | *R^2^* |
| 1. Positive protest tactics | -.14 | .02 | -6.38 | .11 |
| 2. Negative protest tactics | .08 | .03 | 3.15 | .03 |
| 3. Perceived extremity | .18 | .03 | 5.99 | .09 |
| 4. Member support/identification | -.44 | .03 | -14.62 | .38 |
| *Note.* Political ideology was measured on a scale from (1) *Extremely Liberal* to (7) *Extremely Conservative.* | | | | |

| **Table S2. Chi-Square Tests for Participants’ Estimated Frequencies of Events in the Video Clip**  **(Three Category Outcome Measure)** | | | | | | | |
| --- | --- | --- | --- | --- | --- | --- | --- |
| Estimated Frequencies on  Count Variables | None | | 1-10 | | 11-Highest | |  |
| Supporters | Trump  % | Other  % | Trump  % | Other  % | Trump % | Other  % | Chi-Square |
| **Negatively-valenced false events** | | | | | | | |
| 1. Wearing masks | 72.5 | 78.5 | 23.9 | 18.6 | 3.7 | 2.9 | X^2^ (2) = 1.53, ϕ = .07, *p* = .466 |
| 2. Burning things | 86.2 | 94.2 | 12.8 | 5.4 | .9 | .4 | X^2^ (2) = 6.31, ϕ = .13, *p* = .043* |
| 3. Breaking windows | 86.2 | 94.6 | 12.8 | 5 | .9 | .4 | X^2^ (2) = 7.15, ϕ = .14, *p* = .028* |
| 4. Smoking marijuana | 90.8 | 95 | 8.3 | 4.1 | .9 | .8 | X^2^ (2) = 2.51, ϕ = .09, *p* = .285 |
| 5. Exposed breasts | 90.7 | 97.5 | 9.3 | 2.5 | 0 | 0 | X^2^ (2) = 7.87, ϕ = .15, *p* = .005* |
| 6. Fights or brawls | 88.1 | 95 | 11.9 | 4.5 | 0 | .4 | X^2^ (2) = 6.83, ϕ = .14, *p* = .033* |
| 7. Signs “burn it down!” | 80.7 | 87.2 | 19.3 | 12 | 0 | .8 | X^2^ (2) = 4.07, ϕ = .11, *p* = .131 |
| 8. Mexican flags | 80.7 | 88.4 | 19.3 | 11.2 | 0 | .4 | X^2^ (2) = 4.58, ϕ = .11, *p* = .101 |
| 9. Signs misspelled | 63.3 | 79.7 | 36.7 | 19.5 | 0 | .8 | X^2^ (2) = 12.53, ϕ = .19, *p* = .002* |
| **Neutral false events** | | | | | | | |
| 1. Pets or animals | 86.2 | 86.8 | 13.8 | 13.2 | 0 | 0 | X^2^ (2) = .019, ϕ = .01, *p* = .891 |
| 2. Signs w/ cartoons | 43.5 | 43 | 51.9 | 53.3 | 4.6 | 3.7 | X^2^ (2) = .190, ϕ = .02, *p* = .909 |
| **Actual events** |  |  |  |  |  |  |  |
| 1. Pink ‘pussy’ hats | 37.6 | 31.8 | 31.2 | 33.9 | 31.2 | 34.3 | X^2^ (2) = 1.13, ϕ = .06, *p* = .567 |
| 2. Trump references | 52.3 | 40.1 | 45.9 | 53.3 | 1.8 | 6.6 | X^2^ (2) = 6.71, ϕ = .14, *p* = .035* |
| 3. Signs w/ curse words | 56.9 | 66.9 | 37.6 | 31.8 | 5.5 | 1.2 | X^2^ (2) = 7.27, ϕ = .14, *p* = .026* |
| 4. American flags | 55 | 45.5 | 45 | 51.7 | 0 | 2.9 | X^2^ (2) = 5.26, ϕ = .12, *p* = .072 |
| *Table Notes*. The dependent variables were recoded such that values of 0 =0, values 1-10 = 1, values 11 to greatest = 2.  **p* < .05. Chi-square analyses test differences between Donald Trump supporters and other supporters. | | | | | | | |

**Alternative Mediation Models**

Hayes’ (2017) PROCESS Model 4 was used to test the parallel mediating effects of negative and positive protest tactics on perceived extremity of the protest event. Donald Trump supporters perceived more negative protest tactics and fewer positive protest tactics, which in turn predicted increased perceptions of extremity. After accounting for both mediators, voter status remained a significant predictor of perceived extremity, but was reduced, consistent with partial mediation. Indirect effects were tested using a bootstrap estimation approach with 5,000 samples. The indirect effect of voter status on perceived extremity through positive protest tactics was significant, *b* = .04, *SE* = .03, 95% CI [.01, .11]. The indirect effect of voter status on perceived extremity through negative protest tactics was significant, *b* = .27, *SE* = .08, 95% CI [.13, .44]. Thus, for Trump supporters, extremity perceptions were partially mediated through perceiving more negative and fewer positive protest tactics, which increased perceptions of extremity (See Figure S1).

Hayes’ (2017) PROCESS macro Model 6 was used to test the serial mediating effects of perceived negative tactics and perceived extremity in the relation between voter status and support for the cause. Indirect effects were tested using a bootstrap estimation approach with 5,000 samples. The indirect effect of voter status on support for the movement through perceiving negative protest tactics and perceived extremity was significant, *b* = -.07, *SE* = .03 95% CI [-.13, -.03], illustrating that for Trump supporters, support for the movement was partially mediated and reduced through perceiving a greater number of negative protest tactics and greater perceived extremity of the protest event (See Figure S2).

**Variable Guide**

*This document contains variables names and descriptions for all analyses.*

1. polID = continuous measure of political ideology

2. PositiveTactics_mean = mean of 4 items assessing the extent to which protesters used positive protest tactics like expressing positive emotion

3. NegativeTactics_mean = mean of 5 items assessing the extent to which protesters used positive protest tactics like expressing negative emotion

4. BehExtreme = single item assessing perceptions of extremity of the protesters’ behaviour

5. SupportID_mean = mean of 5 items assessing the extent to which participants support the protesters and the movement

6. NegFalse_SUM = sum of 9 items assessing number of objectively false negative events such as wearing masks and burning things

7. NegNeutral_SUM = sum of 2 items assessing number of objectively false neutral events such as pets

8. TrueEvents_SUM = sum of 4 items assessing number of objectively true events such as pink ‘pussy’ hats

9. MEAN.mismemory.R = objectively false negative events such as wearing masks and burning things

10. Count variables recoded into binary outcomes: 1 = incorrect, 0 = correct

Recall_items_masks.Rcorrect

Recall_items_burning.Rcorrect

Recall_items_breakwindows.Rcorrect

Recall_items_marji.Rcorrect

Recall_items_breasts.Rcorrect

Recall_items_fights.Rcorrect

Recall_items_signsburnitdown.Rcorrect

Recall_items_Mxflags.Rcorrect

Recall_items_signsepelling.Rcorrect

Recall_items_pets.Rcorrect

Recall_items_signcartoons.Rcorrect

Recall_items_pussyhats.Rcorrect

Recall_items_signsrefDT.Rcorrect

Recall_items_signcurses.Rcorrect

Recall_items_USflags.Rcorrect

11. Count variables Winsorized:

Recall_items_masksw

Recall_items_burningw

Recall_items_breakwindowsw

Recall_items_mariw

Recall_items_breastsw

Recall_items_fightsw

Recall_items_signsburnitdownw

Recall_items_Mxflagsw

Recall_items_signsspellingw

Recall_items_petsw

Recall_items_signscartoonsw

Recall_items_pussyhatsw

Recall_items_signsrefDTw

Recall_items_signscursew

Recall_items_USflagsw

12. Count variables recoded into trichotomous outcomes (0 = 0, 1-10 = 1, 11 to the highest value= 2)

Recall_items_masks.R

Recall_items_burning.R

Recall_items_breakwindows.R

Recall_items_marji.R

Recall_items_pussyhats.R

Recall_items_breasts.R

Recall_items_USflags.R

Recall_items_Mxflags.R

Recall_items_pets.R

Recall_items_fights.R

Recall_items_signcurses.R

Recall_items_signcartoons.R

Recall_items_signsrefDT.R

Recall_items_signsepelling.R

Recall_items_signsburnitdown.R
